# Tsetse Flies Rely on Symbiotic *Wigglesworthia* for Immune System Development

**DOI:** 10.1371/journal.pbio.1001070

**Published:** 2011-05-31

**Authors:** Kira Heller

**Affiliations:** Freelance Science Writer, Oakland, California, United States of America

**Figure:**
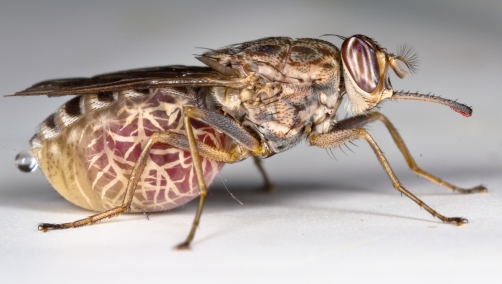
Female tsetse flies provide their intrauterine larvae with nourishment and vertically transmitted symbionts, including obligate *Wigglesworthia*. When this symbiont is absent, tsetse flies exhibit a highly compromised immune system. Image courtesy of Dr. Geoff Attardo.

When we even bother to think about them, we usually regard the bacteria and other microbes that live in our guts and on our skin with disgust. However, recent discoveries about the roles the trillions of microbes living in the average person's gut play in obesity, cardiovascular disease, and immunity have led us to look upon our omnipresent bacterial symbionts with a less jaundiced eye. Mutually beneficial partnerships with bacteria are widespread throughout the animal kingdom: some types of squid have bioluminescent bacteria that help them to escape detection, while marine tubeworms harbor bacteria that provide their hosts with nutrients.

A particularly fascinating example of symbiosis occurs between the tsetse fly *Glossina morsitans* and the bacterium *Wigglesworthia glossinidia* (named after Sir Vincent Wigglesworth, the entomologist with the best name ever). Tsetse flies are the sole vectors for the trypanosomes that cause about 10,000 new cases of sleeping sickness in Africa each year.

*Wigglesworthia*, which can only survive inside the gut of tsetse flies, has a minimal genome, since it lost much of its DNA as it coevolved with its tsetse host over the last 50–80 million years. These bacteria hang out exclusively within tsetse flies; the set-up provides the *Wigglesworthia* with protection and an energy source. In return, the bacteria perform a variety of services for their host, including vitamin synthesis and resistance to energetically costly trypanosome infections, both of which may be important for tsetse fly fertility.

Previously, researcher Serap Aksoy and her colleagues discovered that tsetse flies that lack *Wigglesworthia* are more susceptible to infection with trypanosomes. In a new study described in this issue of *PLoS Biology*, Brian Weiss, Jingwen Wang, and Serap Aksoy explored how *Wigglesworthia* might affect the immune responses of tsetse flies. To do this, they produced larvae that lack *Wigglesworthia* (*Gmm^Wgm−^*) by feeding the antibiotic ampicillin to pregnant female flies (ampicillin selectively kills *Wigglesworthia* without affecting other kinds of bacteria living in the flies). As a handy challenge to the flies' immune system, they injected the flies with *E. coli* K12 bacteria. While mature adult (8-day-old) wild-type (WT) tsetse flies are resistant to infection with *E. coli,* young (3-day-old) flies are quite susceptible. Compared to mature adult WT tsetse, after injection with *E. coli,* the age-matched *Gmm^Wgm−^* dropped like . . . flies.

To figure out the basis of the *Gmm^Wgm−^* flies' compromised immunity, the authors examined the expression of genes known to be involved in immune responses. They found that expression of these genes was virtually the same in uninfected WT and *Gmm^Wgm−^* adults. However, when the flies were injected with *E. coli*, expression increased dramatically in WT flies compared to *Gmm^Wgm−^*. Most striking was the difference in expression of genes involved in cellular immunity processes such as phagocytosis (engulfment of pathogens by host hemocytes) and melanization (laying down of melanin to form a clot at wound sites).

This led the authors to question whether adult WT flies would become more susceptible to infection when phagocytosis was blocked, so they injected tiny beads directly into the circulatory system of mature WT flies in order to divert the hemocytes and make them unavailable for phagocytosis. The bead-injected WT flies turned out to be highly susceptible to *E. coli* infection, indicating that phagocytosis is an important component of the flies' immune response.

Next, to find out if *Wigglesworthia* is necessary for melanization, the authors looked at the sites of *E. coli* injection. In *Gmm^Wgm−^*, the wound was still oozing hemolymph (insect blood) 30 minutes after the injection and no melanin was observed. Meanwhile, in WT flies there was no hemolymph visible and a melanin clot had formed at the wound.

Hemocytes play a central important role in cellular immunity; not only do they phagocytose pathogens, but differentiated hemocytes called crystal cells also produce clot-forming melanin. Counting circulating and sessile hemocytes revealed that adult *Gmm^Wgm−^* had far fewer hemocytes than their wild-type counterparts. The authors speculated that the absence of hemocytes in adult *Gmm^Wgm−^* flies reflects a lack of blood cell differentiation during development. This was borne out by the drastically decreased expression in *Gmm^Wgm−^* of two transcription factors known to be involved in hemocyte differentiation in *Drosophila.*

Together, these results show that *Wigglesworthia* must be present in immature tsetse flies so that the immune system can develop and function properly in adults. Thus, reminiscent of the relationship between humans and the bacteria in their guts, *Wigglesworthia* and tsetse flies have coevolved to the point where they can't really survive without each other. The *Wigglesworthia–*tsetse fly association is a great model system for studying the effect of symbionts on host immunity, because of the short generation times of the flies, which are easy and inexpensive to rear. Furthermore, the authors' findings could lead to new ways of modulating tsetse flies' immune response to trypanosomes, hopefully making them more resistant to infection and therefore less efficient vectors for these deadly pathogens.

**Weiss BL, Wang J, Aksoy S (2011) Tsetse Immune System Maturation Requires the Presence of Obligate Symbionts in Larvae. doi:10.1371/journal.pbio.1000619.**

